# Interferon-β Stimulation Elicited by the Influenza Virus Is Regulated by the Histone Methylase Dot1L through the RIG-I-TRIM25 Signaling Axis

**DOI:** 10.3390/cells9030732

**Published:** 2020-03-16

**Authors:** Laura Marcos-Villar, Estanislao Nistal-Villan, Noelia Zamarreño, Urtzi Garaigorta, Pablo Gastaminza, Amelia Nieto

**Affiliations:** 1 Departamento de Biologia Molecular y Celular, Centro Nacional de Biotecnología, C.S.I.C. Darwin 3, Cantoblanco, 28049 Madrid, Spain; nzamarre@cnb.csic.es (N.Z.); ugaraigorta@cnb.csic.es (U.G.); pgastaminza@cnb.csic.es (P.G.); 2 Ciber de Enfermedades Respiratorias CIBERES, 28029 Madrid, Spain; 3 Microbiology Section, Dpto. CC, Farmacéuticas y de la Salud, Facultad de Farmacia, Universidad CEU San Pablo, CEU Universities, Boadilla del Monte, 28660 Madrid, Spain; estanislao.nistalvillan@ceu.es; 4 Instituto de Medicina Molecular Aplicada (IMMA), Universidad CEU San Pablo, Pablo-CEU, CEU Universities, Boadilla del Monte, 28660 Madrid, Spain

**Keywords:** Dot1L histone methylase, TRIM25, influenza virus, RIG-I signaling, Type I IFN, RNA virus infection

## Abstract

Influenza virus infection increases the methylation of lysine 79 of histone 3 catalyzed by the Dot1L enzyme. The role of Dot1L against infections was highlighted by an increase of influenza A and vesicular stomatitis virus replication in Dot1L-inhibited cells mediated by a decreased antiviral response. Interferon-beta (IFN-β) reporter assays indicate that Dot1L is involved in the control of retinoic acid-inducible gene-I protein (RIG-I) signaling. Accordingly, Dot1L inhibition decreases the IFN-β promoter stimulation and RIG-I- mitochondria-associated viral sensor (RIG-I-MAVS) association upon viral infection. Replication of an influenza A virus lacking NS1 (delNS1), incapable of counteracting the antiviral response, is not affected by Dot1L inhibition. Consequently, RIG-I-MAVS association and nuclear factor–κB (NF-κB) nuclear translocation, are not affected by the Dot1L inhibition in delNS1 infected cells. Restoration of NS1 expression in *trans* also reinstated Dot1L as a regulator of the RIG-I-dependent signaling in delNS1 infections. Interferon-inducible E3 ligase tripartite motif-containing protein 25 (*TRIM25*) expression increases in influenza virus infected cells, but Dot1L inhibition reduces both the *TRIM25* expression and TRIM25 protein levels. *TRIM25* overexpression reverses the defective innate response mediated by Dot1L inhibition elicited upon virus infection or by overexpression of RIG-I signaling intermediates. Thus, TRIM25 is a control point of the RIG-I recognition pathway controlled by Dot1L and may have a general role in RNA viruses recognized by the RIG-I sensor.

## 1. Introduction

Epigenetic changes in host cells induced by infection of the influenza virus have been poorly characterized. Infection with the highly pathogenic H5N1 virus generated changes in the total DNA methylation in the thymus of infected chickens [1] and in the methylation of several inflammatory genes in human lung epithelial A549 cells [2,3]. Changes in the histone methylation status of several interferon-stimulated genes (ISGs) in human respiratory cells infected with an H1N1 strain were also reported [4]. Performing an unbiased screening of epigenetic changes in A549 influenza H1N1 virus-infected cells, we observed unaltered DNA methylation and a general decrease in histone acetylation, which correlates with the cell host transcriptional inactivation that takes place during infection.

Unexpectedly, we observed an increase in the methylation of lysine 79 of histone 3 (H3K79) in the infected cells [5]. The methylation of H3K79 is carried out by the histone methylase Dot1L, which exclusively methylates this lysine, and its methylation is increased in actively transcribing genes [6,7]. The inhibition of Dot1l by pinometostat (EPZ) treatment or by Dot1L silencing, stimulated influenza and vesicular stomatitis virus replication [5]. Further characterization showed decreased nuclear translocation of the NF-kB complex, as well as IFN-β, Mx1 and ISG56 expression in EPZ-treated cells [5] indicating a decreased antiviral type I IFN response, and as a consequence, a stimulation in viral replication. Conversely, Dot1L overexpression reduced the multiplication of the virus [8].

Invading viral RNAs are detected by different cytoplasmic sensors that promote type I IFN responses to elicit an antiviral state. Among the cytoplasmic RNA sensors, RIG-I plays a pivotal role in the recognition of negative-strand RNA viruses [9,10]. The melanoma differentiation-associated protein 5 (MDA5), has been involved in the recognition of the RNA of picornaviruses [11]. However, some viruses appear to be sensed by both RIG-I and MDA5 [12]. After RNA binding, RIG-I and MDA5 undergo conformational changes that release the tandem caspase activation and recruitment domains (2CARDs). After the recruitment of different phosphatases, the phosphorylation marks of the 2CARDs domains are removed [13].

In the case of the CARD domains of RIG-I, ubiquitination by TRIM25 allows for interaction with MAVS, the adaptor protein located at the mitochondrial membrane [14,15]. The multimerization of MAVS triggers the formation of the IKKα–IKKβ–IKKγ ternary complex that induces the nuclear translocation of NF-κB and the formation of the TBK1 and IKKε complex, which activates the IFN regulatory factor 3 (IRF3) and/or IFN regulatory factor 7 (IRF7) [16]. The activation of NF-κB, IRF3, and IRF7 stimulates the transcription of inflammatory cytokines and interferon genes, eliciting the expression of many ISGs and creating an antiviral state (see for review [17]).

Here, we analyzed which steps of the type I IFN pathway are affected by Dot1L to control the innate immune response. The inhibition of Dot1L decreases the TRIM25 expression, the association of RIG-I-MAVS at the mitochondria, and the NF-κB and IRF-3 activation in influenza virus infected cells through the RIG-I signaling axis. The overexpression of TRIM25 counteracts the effects of Dot1L inhibition on influenza virus infection and RIG-I signaling, indicating that TRIM25 is a target of regulation of Dot1L histone methylase.

## 2. Materials and Methods

### 2.1. Plasmids

The IFN-β-luciferase reporter IRF-3-responsive plasmid (p55-IRF3), the plasmid expressing the constitutively active phosphomimetic IRF3 mutant (pCAGGS-hIRF3 5D), and the plasmids encoding the wild type RIG-I CARDS (pCAGGS-flag-hRIG-I CARD210) or the RIG-I CARDS with a substitution of Ser 8 with phosphomimetic aspartate (which results in decreased TRIM25 binding and RIG-I ubiquitination (pCAGGS-flag-hRIG-I S8D)) were previously reported [18,19,20,21]. The following plasmids were acquired from commercial companies: the plasmid expressing Renilla luciferase (pRenillaLuc-null) from Clontech (Takara Holdings Inc. Shimogyō-ku, Kyoto, Japan), the plasmid that expresses RIG-I (pEF-BOS RIG-I MIII) and the plasmid expressing MDA5 (pEF-BOS MDA5) from Addgene (Watertown, Massachusetts, USA), the plasmid that expresses luciferase under the NF-κB promoter (pNF-kB(cis)-LUC) from Agilent (Santa Clara, CA, USA), and the pcDNA3.0 plasmid from Invitrogen (Thermo Fisher Scientific, Waltham, MA, USA). The plasmid that expresses the firefly luciferase gene under the IFN-β promoter, the pIF-LukTer (−116/+72), was kindly provided by Steve Goodbourn (Institute for Infection and Immunity, St. George’s, University of London, London, United Kingdom), the plasmid that expresses MAVS (pEF BOS-MAVS His 6X; FLAG Cterminal) by KA. Fitzgerald (Division of Infectious Diseases and Immunology, University of Massachusetts Medical School, Worcester, MA 01605, USA) and the plasmid expressing TRIM25 (pcDNA3.0-HA- TRIM25) was provided by Carmen Rivas (Centro de Investigación en Medicina Molecular (CIMUS), Universidade de Santiago de Compostela, Santiago de Compostela, Spain)

### 2.2. Cells and Viruses

Human respiratory cells (A549), Madin-Darby canine kidney (MDCK), and MDCK expressing NS1 (MDCK-NS1) cells [22] were cultured in Dulbecco’s modified Eagle’s medium (DMEM) with 10% fetal calf serum. The mouse-adapted influenza A/PR78/34 (PR8) and delNS1 [23] viruses were used. Vesicular stomatitis virus (VSV) was provided by R. Alfonso, and Sendai virus was provided by P. Gastaminza. EPZ5676 (EPZ) was purchased from Novagen (Merck KGaA. Darmstadt, Germany) and human tumor necrosis factor α (TNFα) from Sigma-Aldrich (Merck KGaA (Darmstadt, Germany).

### 2.3. Virus Infection

Cultured A549, MDCK, or MDCK-NS1 cells were infected at 1–3 pfu/cell (high MOI) or at 1 × 10^−3^ PFU/cell (low MOI) with the corresponding virus as indicated in each figure legend. After 1–2 h, non-bound virus was rinsed off and, at different times (hours post-infection; hpi), the cell extracts were collected and used for virus titration by plaque assay or for Western blot. Cell culture infections were performed in BSL2 conditions.

### 2.4. Reporter Assays

A549 cells were plated with or without 1 μM EPZ (96-well plates). Forty-eight hours later the cells were transfected with a total of 40 ng per well of reporter plasmids together with 4 ng of Renilla luciferase expressing plasmid for normalization (Viromer RED one, Weinbergweg, Saale, Germany). In the cotransfection experiments, equal amounts of cotransfected plasmids were used, and the pcDNA3.1 plasmid was used as an empty vector to compensate the total amount of DNA per well. Depending on the experiment, 16–24 h later the cells were collected and used for luciferase detection or were infected and, at 16 hpi, used for luciferase assay.

In the silencing experiments, A549 cells were plated and transduced with the corresponding lentiviruses at the same time as plating. Twenty-four hours later, 50 µg/mL of puromycin was added, and 5 days later they were plated in 96-well plates. Once the cells were attached to the plate (around 8 h later), they were transfected and processed as indicated above.

The detection of firefly and Renilla luciferase activities was performed using the Dual-Luciferase^®^ Reporter Assay Kit, Promega Biotech Iberica, Madrid, Spain). Depending on the experiment, 16–32 h after transfection, the cells were harvested and lysed in dual reporter lysis buffer (Promega). Luciferase activity was normalized by Renilla luciferase in each condition and the value obtained in control cells was set as 100%. When using lentiviruses, the luciferase results (previously normalized by Renilla luciferase) were expressed as the fold change relative to each control condition.

### 2.5. Statistical Analysis

Statistical analysis was performed using PRISM version 5.0 (GraphPad Software, San Diego, CA, USA). The data are presented as the mean ± SD. The comparisons between two groups were made using a two-tailed unpaired *t*-test. Multiple groups were compared using ANOVA followed by a Bonferroni post-test. Statistical significance was assigned to *p*-values less than 0.001 (***), less than 0.01 (**), or less than 0.05 (*).

### 2.6. Western Blot

Viral proteins were detected using in-house produced rat polyclonal antibodies against NP protein (1:5000; [24]) and against NS1 protein [25]. Cellular proteins and their modifications were detected using commercially available antibodies as follows: rabbit polyclonal antibodies to H3K79me2 (D15E8), H3 (D1H2), and CHD1a (D8C2) (1:1000) from Cell Signaling (Danvers, MA, USA); rabbit polyclonal antibodies anti-TRIM25 (EPR7315) (ab167154), anti-RIG-I (DDX58) (ab45428), and anti-Ubiquitin UbK63 (linkage-specific K63) (EPR8590-448) (ab179434) from Abcam (Cambridge, United Kingdom); mouse monoclonal antibodies anti-MAVS (E-3 (sc-166583); 1:1000), and anti-β-tubulin (1:1000) from Santa Cruz (Dallas, TX, USA) and Sigma-Aldrich, respectively.

### 2.7. Colorimetric Determination of H3K79me2

The iQuik Global Di-Methyl Histone H3K79 Quantification Kit (Colorimetric) from Epigentek (Farmingdale, NY, USA) was used for ELISA-like measurement of total H3K79me2 amounts, following the supplier’s protocol (Catalog # P-3056).

### 2.8. Confocal Immunofluorescence Microscopy

Cells were fixed in 4% paraformaldehyde (10 min, room temperature) and stored in PBS. For immunofluorescence, the cells were permeabilized (10 min) in PBS containing 1% Triton X-100 and incubated with primary antibodies diluted in PBS/4% BSA (*w*/*v*) as follows: rat anti-NP (1:5000; [24]), rabbit anti-p65 (ab16502) (1:500; Abcam, Cambridge, United Kingdom and mouse anti-MAVS antibody (E-3): sc-166583 (1:500, Santa Cruz, Dallas TX, USA)). Confocal microscopy was performed with a Leica TCS SP5 laser scanning system (Leica Microsystems, Wetzlar, Germany). Images of 1024 × 1024 pixels with eight-bit grayscale depth were acquired sequentially every 0.2–0.3 μm using LAS AF version 2.2.1 software (Leica) and analyzed using LAS AF and MetaMorph Premier version 7.5.2 image analysis software (Molecular Devices, ForteBio, Wokingham, Berkshire UK).

### 2.9. Coimmunoprecipitation Assays

Immunoprecipitation studies were performed as described previously [26]. We plated 10^7^ A549 cells and left them untreated or treated with 1 μM EPZ, and 48 h later the cells were mock-infected or infected with influenza virus PR8 or PR8 deltaNS1 at 3 MOI. At 16 hpi, the cells were collected and lysed in buffer containing 150 mM NaCl, 5 mM EDTA, 1.5 mM MgCl_2_, 50 mM Tris/HCl pH 8.5, and 0.5% Igepal, with “complete” protease inhibitors (Roche, Basilea, Switzerland). The lysate was centrifuged at 10,000× *g* and the supernatant was used for immunoprecipitation studies using 1 µg of the MAVS antibody for each condition. The immunocomplexes recovered were washed 10 times with lysis buffer and the immunoprecipitated proteins were analyzed by Western blots.

### 2.10. Mitochondria Purification

Mitochondrial fractions from the A549 cells were isolated using a Mitochondria Isolation Kit (ab110170) (Abcam, Cambridge, United Kingdom) following the manufacturer’s instructions.

### 2.11. qRT-PCR Analysis

For RNA extraction, the cell pellets were resuspended in 1 mL TRIzol reagent (Invitrogen) and the RNA was purified as recommended by the manufacturer. The RNA was digested with RNAse-free DNAse (1 U/mg; 1 h, 37 °C), extracted with phenol-chloroform-isoamyl alcohol, and ethanol-precipitated.

For reverse transcription, we used the High-Capacity cDNA RT kit (Applied Biosystems-Thermo Fisher Scientific Waltham, MA, USA). PCR was performed in 96-well PCR plates using the SYBR green PCR master mix (Applied Biosystems). PCR was carried out in a PRISM 7500 Sequence detection system (Applied Biosystems). The cycle threshold (Ct) was determined with analytical software (SDS; Applied Biosystems). Serial dilutions of cDNA were used to ensure amplification.

### 2.12. RNA seq

The total RNAs were isolated and used for high-throughput sequencing with TruSeq v3 chemistry (San Diego, CA 92121, USA) and 50 bp single reads on an Illumina HiSeq 2000. For RNA-seq analysis, sequenced reads were aligned to the Homo sapiens genome (version GRCh38.p8 from NCBI) using TopHat 2.1.1 [27] linked to Bowtie 2.2.8 [28] with the default sensitive settings. From the sequenced reads, transcripts were assembled using Cufflinks 2.2.1 [29]. Differential expression analyses were performed with Cuffdiff 2.2.1 [30] using a minimum false-discovery rate (qvalue) of <0.002 as statistical significance and log2 fold change >1.5 as the cut-off. Additional information for each gene was obtained from the NCBI database and included in the dataset. Genes with expression levels under a threshold in both the control and treatment conditions were discarded. The median of the distribution of the non-zero values was taken as the threshold. The generated data were uploaded to the FIESTA viewer FIESTA@BioinfoGP (https://bioinfogp.cnb.csic.es/tools/FIESTA/index.php)

## 3. Results

### 3.1. Inactivation or Downregulation of Dot1L Inhibited IFN-β Promoter Stimulation in Influenza, Sendai, and Vesicular Stomatitis Virus Infection

**Figure 1:**
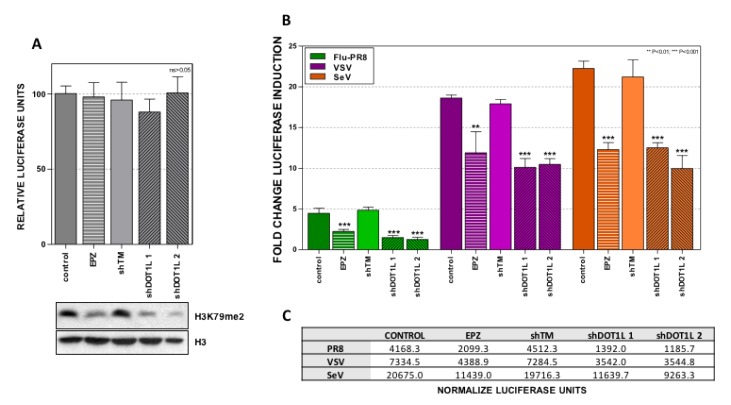
Dot1L inhibition or downregulation decreases IFN-β promoter stimulation in infected cells. (**A**) Upper part: A549 cells were left untreated (control), treated with a Dot1L inhibitor (EPZ, 1 μM, 48 h) or infected with lentiviruses expressing an irrelevant shRNA (shTM) or specific Dot1L shRNAs (shDOT1L 1 and shDOT1L 2) for 5 days. The cells were then transfected with plasmid pIF-LukTer and the luciferase activity was evaluated 16 h later. The luciferase activity was normalized by Renilla luciferase and the value obtained in the control cells was set as 100%. Lower part: Total extracts were used for Western blot analysis against the indicated proteins. (**B**) A549 cells were left untreated (control), treated with Dot1L inhibitor (EPZ, 1 μM, 48 h), or infected with lentiviruses expressing the indicated shRNAs for 5 days. The cells were then transfected with plasmid pIF-LuKter and 24 h later they were infected with influenza (FLU-PR8), or Sendai (SeV), or vesicular stomatitis virus (VSV) at MOI of 1. The luciferase activity was measured at 16 hpi and it is shown as the fold change relative to the levels in each control condition after normalization with the Renilla luciferase levels in each sample. The relative luciferase units obtained in each condition are represented in the lower part. Three technical replicates of three independent experiments were analyzed. ns *p* > 0.05; * *p* < 0.05; ** *p* < 0.01; *** *p* < 0.001.

To characterize which steps of the cascade, involved in the type I IFN stimulation, were modulated by Dot1L, we set up a transfection system, which uses cDNA expression plasmids of well-characterized genes involved in the different steps. First, the A549 cells were left untreated (Control) or treated with Dot1L inhibitor EPZ (1 μM), 48 h later the cells were transfected with a plasmid that expresses luciferase under the interferon beta promoter (pIF-LukTer), and at 24 h post-transfection (hpt) luciferase activity was evaluated. Transfection with pIF-LukTer triggered a basal luciferase expression that was not altered when inhibiting Dot1L (Figure 1A). In addition, the downregulation of Dot1L expression was also performed using lentiviruses expressing shRNA specific for Dot1L (shDot1L.1, shDot1L.2) [5] or a control that expresses irrelevant shRNA (shTM) [31,32].

A549 cells were infected with these lentiviruses, and 5 days later they were transfected with pIF-LukTer. The luciferase activity was examined at 24 hpt as described in Section 2 (Figure 1A). In all the transfection experiments, a plasmid expressing Renilla luciferase under a minimal constitutively active promoter (pRenillaLuc-null) was added to the transfection mixture for normalization. In agreement with the effect caused by the chemical inhibition of Dot1L, the downregulation of Dot1L did not change the luciferase expression. The inhibition and downregulation of Dot1L were indirectly confirmed by analyzing the accumulation of H3K79me2, the target of Dot1L, by Western blot.

We previously described the inhibition of the innate immune response against influenza virus infection by Dot1L inhibition [5]. To validate the transfection system upon viral infection, A549 cells were left untreated or treated with Dot1L inhibitor EPZ. The cells were transfected with pIF-LukTer 48 h later and were subsequently infected with influenza (FLU-PR8), Sendai (SeV), or vesicular stomatitis virus (VSV) at high MOI 24 h post-transfection (hpt), and the luciferase activity was evaluated at 16 hpi (Figure 1B). The activation of luciferase activity was observed upon infection with all three viruses. The decreased stimulation of luciferase was observed upon EPZ treatment in all conditions, confirming that Dot1L inhibition reduces the innate immune activation.

In parallel experiments, the A549 cells were transduced with the different lentiviral vectors, followed by transfection with pIF-LukTer and processed as described above. A similar decrease of luciferase activity was found in downregulated or inhibited Dot1L cells. Collectively, these results indicate that the transfection system recapitulated the effect of Dot1L inhibition on the innate immune response in infected cells [5] and suggest that Dot1L histone methylase modulates a common step of the interferon pathway that controls the antiviral response to different RNA viruses.

### 3.2. Dot1L Controls NF-κB and IRF3 Activation

**Figure 2:**
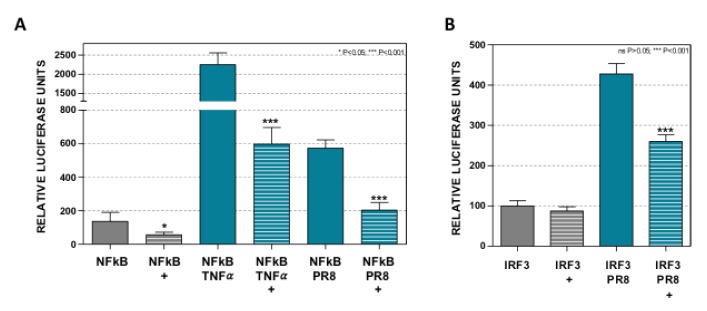
Dot1L controls NF-κB and IRF3 activation. A549 cells were left untreated or treated with 1 μM EPZ (+), 48 h later the cells were (**A**) transfected with a plasmid expressing luciferase under the NF-κB promoter (NF-κB) and at 24 hpt they were stimulated with TNF-α β20 ng/mL, NF-κB-TNFα), or infected with influenza virus (PR8, MOI 1, NF-κB-PR8), and the luciferase activity was evaluated at 16 h later; (**B**) transfected with reporter plasmid p55-IRF3 expressing luciferase under the IRF3 promoter, and the luciferase activity was evaluated 16 h later. In all cases, the luciferase activity values were normalized by Renilla luciferase and they were expressed relative to their corresponding EPZ untreated control conditions. Three technical replicates of three independent experiments were analyzed. ns *p* > 0.05; * *p* < 0.05; ** *p* < 0.01; *** *p* < 0.001.

Antiviral response is stimulated by the activation of NF-κB, IRF3, and/or IRF7 during infections. We have previously described that the inhibition of Dot1L caused a significant reduction of NF-κB nuclearization and IFN-β expression upon influenza virus infection or TNF-α treatment [5]. The reporter plasmid used to monitor IFN-β promoter stimulation (Figure 1) includes NF-κB and IRF3 binding sites. To analyze whether Dot1L regulates the NF-κB and/or IRF3 activation in the transfection system, EPZ-treated (+) or untreated A549 cells were transfected with a NF-κB promoter dependent luciferase expressing plasmid (NF-κB). The transfected cells were then stimulated with TNF-α (NFκB−ΤΝFα) or they were infected with influenza virus at MOI 1 (NFκB-PR8) (Figure 2A). High stimulation of the NF-κB-driven expression of luciferase was observed in TNF-α and influenza virus infected cells, and in accordance with our previous data [5], a significant reduction was observed by Dot1L inhibition, even in the absence of external stimuli (Figure 2A).

Untreated and EPZ-treated A549 cells were also transfected with a plasmid expressing luciferase under the IRF3 promoter (p55-IRF3) and the cells were left uninfected or infected with PR8 at MOI 1 (Figure 2B). A significant stimulation of the IRF3-driven expression of luciferase was observed in the infected cells that diminished upon Dot1L inhibition. The basal activity of the IRF3-dependent promoter was unaffected by EPZ while the basal activity of the NF-κB-dependent promoter was inhibited by EPZ. These results confirm that NF-κB-dependent stimulation was inhibited by EPZ independently of viral infection [5] but also reveal an additional impact of EPZ on virus-induced IRF3 activation.

### 3.3. Dot1L modulates the antiviral response mediated by RIG-I signaling

The above data suggested that Dot1L may control the antiviral response upstream of the NF-κB and IRF3 signaling steps. Two different RNA sensors trigger the MAVS-CARDS interaction, MDA5 and RIG-I. To explore their contribution, cotransfection experiments of pIF-LucTer together with plasmids expressing components of the two pathways were performed. The coexpression of pIF-LukTer with a plasmid expressing MDA5 (pEF-BOS MDA5) stimulated luciferase activity but this activation was not significantly modified in EPZ treated-cells (Figure S1), suggesting that MDA5 does not play a pivotal role in the Dot1L-mediated regulation of the IFN pathway. As a control, we cotransfected pIF-LukTer with a plasmid that expresses a constitutively active mutant form of the IRF3 protein where key Ser/Thr residues have been substituted by five phosphomimetic aspartic residues (pCAGGS-hIRF3 5D) causing IFN-β promoter activation independently of any stimuli [19]. The EPZ treatment did not modify the luciferase activity levels upon IRF3 mutant coexpression (Figure S1) suggesting that signaling events upstream of IRF3 activation are modulated by EPZ.

**Figure 3:**
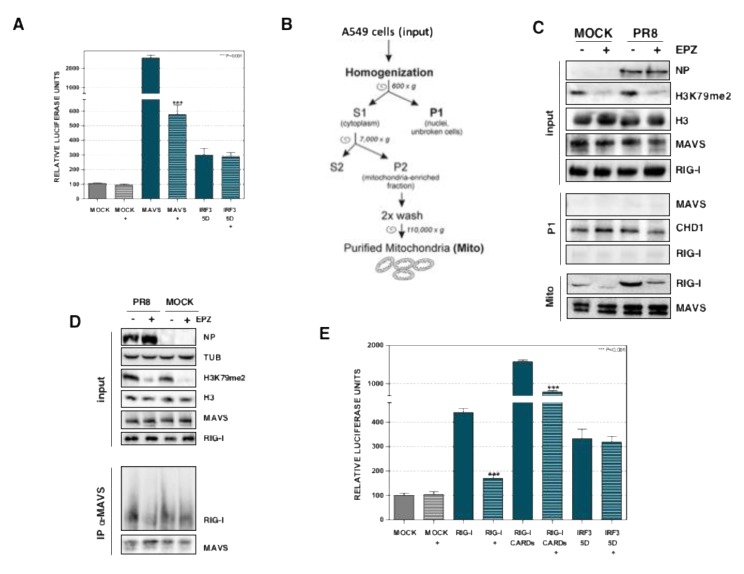
Dot1L modulates MAVS activation, RIG-I-MAVS association, and the antiviral response mediated by the RIG-I sensor. (**A**) A549 cells were left untreated (MOCK) or treated with 1 μM EPZ (+), 48 h later the cells were transfected with pIF-LukTer (MOCK), or cotransfected with pIF-LukTer together with plasmids expressing MAVS (MAVS), or mutant IRF3 (IRF3 5D) and the luciferase activity was evaluated at 16 hpt. (**B**) Scheme of the mitochondria purification. (**C**) Control or EPZ-treated cells were left uninfected (MOCK), or infected with PR8 virus at MOI 3 for 8 h. Input, P1 (P1), and purified mitochondria (Mito) fractions were used for Western blot analysis against the indicated proteins. (**D**) Control or EPZ-treated cells (1 μM (+), 48 h) were left uninfected (MOCK), or infected with PR8 virus at MOI 3 (PR8). At 16 hpi, the total extract was used for immunoprecipitation analysis using antibodies against MAVS. The immunoprecipitate was used for Western blot analysis against the indicated proteins. (**E**) Control or EPZ-treated cells were transfected with pIF-LukTer (MOCK), or cotransfected with pIF-LukTer, together with plasmids expressing RIG-I (RIG-I), or RIG-I 2CARDs (RIG-I CARDs), or mutant IRF3 (IRF35D) and the luciferase activity was evaluated at 16 hpt. In panels A and E, the luciferase activity was normalized by Renilla luciferase. The MOCK condition without EPZ treatment was taken as 100%. Three technical replicates of three independent experiments were analyzed. ns *p* > 0.05; * *p* < 0.05; ** *p* < 0.01; *** *p* < 0.001.

RIG-I has a pivotal role as a sensor of influenza, and other negative-strand RNA viruses, to promote MAVS-2CARDs interactions [9]. The interaction of MAVS with the 2CARD domains of RIG-I is necessary to allow the formation of the IKKα–IKKβ–IKKγ complex that regulates NF-κB traffic [16] and the TBK1 and IKKε complex that activates the IFN regulatory factor 3. Control or EPZ-treated A549 cells were cotransfected with pIF-LukTer and a plasmid that expresses MAVS (pEF BOS-MAVS), as MAVS overexpression is sufficient to trigger downstream signaling [33]. As expected, a statistically significant increase in luciferase activity was observed in untreated cells upon MAVS overexpression. Importantly, inhibition of Dot1L reduced 5–10 times MAVS-induced IF-β promoter activation (Figure 3A), which agrees with the effect of EPZ in NF-κB-dependent luciferase stimulation (Figure 2A). Transfection of the phosphomimetic IRF3 mutant plasmid was included as a control.

The ubiquitination of the 2CARDs domains of RIG-I promotes RIG-I’s recruitment to the mitochondrial membrane where it interacts with MAVS, triggering the activation of the IFN pathway [34]. Thus, we evaluated whether Dot1L modulates the endogenous RIG-I recruitment to the mitochondria in influenza virus infected cells. The control or EPZ-treated A549 cells, were left uninfected (MOCK) or infected with influenza virus (PR8) at MOI 3. We obtained 8 hpi enriched mitochondrial fractions by successive centrifugation steps (Figure 3B) as described in Section 2, and different fractions were used for Western blot analysis (Figure 3C).

As expected, the detection of NP viral protein in the infected cells and a specific reduction of H3K79 levels in EPZ-treated cells, were found in the input (Input). Undetectable levels of MAVS were present in the P1 pellet (nuclei and unbroken cells), whereas the nuclear chromodomain helicase DNA binding protein 1 (CHD1), was present. In addition, MAVS was clearly detected in the mitochondrial fractions (Mito), indicating a correct isolation of mitochondria. In agreement with the previous reports [35], the recruitment of RIG-I to the enriched mitochondrial fraction was clearly found in the influenza infected cells. We observed a decreased association of RIG-I to the enriched mitochondrial fraction in the Dot1L inhibited cells.

The results suggested that Dot1L inhibition involves a dampening of the ability of RIG-I to translocate to mitochondria and induce MAVS signalosome formation. To verify this issue independently, MAVS aggregation, as well as NF-κB distribution, were analyzed by confocal immunofluorescence microscopy. PR8-infected cells, untreated or EPZ treated (+), were fixed and processed for immunofluorescence microscopy at 8 hpi, as described in Section 2 (Figure S2A). The inhibition of NF-κB nuclear localization (p65) was observed in infected cells treated with the inhibitor as compared with the untreated controls, as previously described [5]. MAVS showed a granular distribution in the cytosol of non-infected cells (Figure S2A; MOCK).

The aggregation of MAVS was readily observed in influenza-infected cells (Figure S2A; PR8) but was not observed in EPZ-treated cells (Figure S2A; PR8+). We performed further analysis to distinguish the MAVS polymerization (Figure S2B). Control or PR8 infected cells, untreated or treated (+) with EPZ, were used for SDD-AGE analysis, which consists of a semi-denaturing detergent agarose gel electrophoresis [36]. High molecular weight aggregates of MAVS were observed in infected cells, especially at 16 hpi, and Dot1L inhibition caused a reduction of these MAVS aggregates, supporting the notion that Dot1l-inhibition interferes with MAVS activation.

Next, coimmunoprecipitation experiments were performed to explore the association of endogenous RIG-I-MAVS protein complexes in untreated or EPZ-treated control or PR8 infected cells (Figure 3D). EPZ-treatment did not decrease the RIG-I-MAVS association in control cells, while a decreased association was found in infected cells.

To examine the relevance of the 2CARDs domain ubiquitination of RIG-I in Dot1L regulation, we performed cotransfection experiments of pIF-LukTer with plasmids that express RIG-I or the 2CARDs domain of RIG-I (RIG-I CARDs) (Figure 3E). The coexpression of RIG-I or RIG-I 2CARDs increased the luciferase activity, and inhibition of Dot1L caused a significant decrease. The relative luciferase units obtained in parts A and E are presented in Figure S3. These results indicate that Dot1L controls RIG-I signaling and MAVS activation, which supports a role of Dot1L in regulating the RIG-I pathway.

### 3.4. The NS1 Protein of Influenza Virus Modulates the Dot1L Effect on Virus Replication

**Figure 4:**
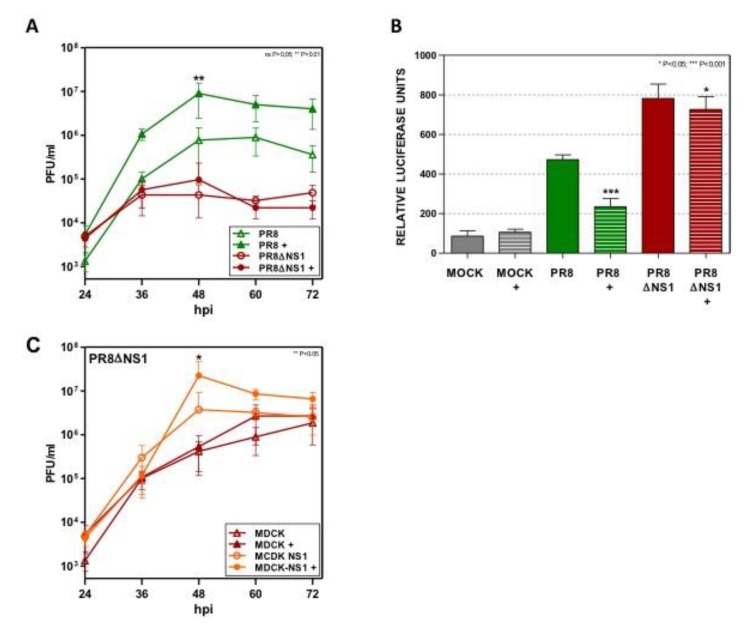
Dot1L does not affect viral replication or IFN-β promoter stimulation in delNS1 virus infected cells. (**A**) A549 cells were plated in the presence or absence of EPZ 1 µM and 48 h later they were infected at MOI 10^−3^ with PR8 or delNS1 (PR8ΔNS1). The virus titer was determined with a plaque assay on MDCK cells. (**B**) A549 cells were left untreated or treated with 1 μM Dot1L inhibitor (+) and 48 h later they were transfected with the plasmid pIF-LukTer. At 24 h post-transfection, the cells were left uninfected (MOCK), or infected with influenza virus (PR8) or delNS1 virus (PR8ΔNS1) at MOI 1 and the luciferase activity was evaluated 16 h later. The luciferase activity normalized by Renilla luciferase in the MOCK condition without EPZ treatment was taken as 100%. (**C**) MDCK or MDCK-NS1 cells were plated in the presence or absence of EPZ 1 µM and 48 h later they were infected at MOI 10^−3^ with delNS1. The virus titer was determined by plaque assay on MDCK cells. EPZ was present all throughout the experiment in the EPZ-treated cells. Three technical replicates of three independent experiments were analyzed. ns *p* > 0.05; * *p* < 0.05; ** *p* < 0.01; *** *p* < 0.001.

The influenza virus expresses the NS1 protein; a multifunctional protein that modulates viral replication and host cell physiology [37]. The main activity of NS1 in the influenza virus life cycle has been associated with its ability to inhibit the host immune response, limiting both interferon production and IFN effector function [37]. Accordingly, the production of IFN-β and the activation of STAT1 and STAT2 signaling are inhibited by NS1 in A549 cells infected with the influenza virus [38]. As Dot1L regulates the innate immune response, we analyzed the impact of Dot1L inhibition during infection with a PR8 strain that does not express the NS1 protein (delNS1virus, PR8ΔNS1) [23]. The A549 cells were left untreated or treated with Dot1L inhibitor and infected with the PR8 strain or with delNS1 virus at MOI 10^−3^ (Figure 4). According to the previous results, treatment with EPZ increased the influenza virus replication in A549 cells infected with PR8.

In contrast, a similar replication was found in cells infected with delNS1 virus treated with the inhibitor (Figure 4A). These results suggest that EPZ-mediated virus growth stimulation involves a NS1-related mechanism. Additional experiments in the transfection system were performed in control or EPZ treated cells transfected with pIF-LukTer and infected with PR8 or delNS1 virus (MOI 1, 16 hpi). Both viruses induced IFN-β promoter activity, although with greater intensity in the delNS1, as expected from the role of NS1 on dampening the host innate responses. In contrast to PR8 infection, where EPZ inhibited the IFN-β promoter activation by approximately two-fold, Dot1L inhibition only caused a marginal decrease in the luciferase activity in delNS1 infection (Figure 4B), supporting the notion that NS1 is required to observe the impact of EPZ on the innate responses against the influenza virus.

To demonstrate the role of NS1 on Dot1L-mediated regulation of virus replication, we infected untreated or EPZ-treated parental MDCK cells or MDCK cells that constitutively express NS1 protein (MDCK-NS1) with delNS1 virus at MOI 10^−3^ [22], in order to *trans*-complement delNS1 (Figure 4C). The Dot1L inhibitor did not affect delNS1 replication in MDCK cells, in agreement with the results obtained in the A549 cells (Figure 4A). In contrast, augmented virus replication was found in the MDCK-NS1 cells treated with the Dot1L inhibitor and infected with delNS1 (Figure 4C). These results suggest that NS1 is required to observe the impact of Dot1L inhibition in the control of influenza virus replication. Alternatively, Dot1L may control an element in the IFN-β signaling pathway that is regulated by the influenza virus NS1 protein.

### 3.5. NS1 Protein Modulates the Dot1L Control of RIG-I-MAVS Association and NF-kB Nuclearization

**Figure 5:**
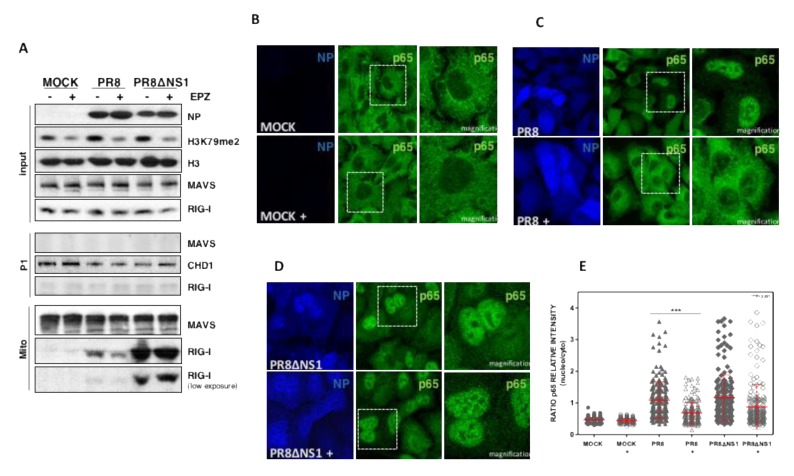
Dot1L inhibition does not modify RIG-I-MAVS interaction and nuclear translocation of NF-κB in delNS1 infection. (**A**) Control or EPZ-treated cells were left uninfected (MOCK), or infected with PR8 or with delNS1 virus at MOI 3. Input, P1 (P1) and purified mitochondria (Mito) fractions were used for Western blot analysis against the indicated proteins. (**B**) A549 cells were plated in the presence or absence of EPZ 1 µM and 48 h later, the cells were processed for immunofluorescence using anti-p65. (**C**,**D**) Cells were processed as in (**B**) and then infected at MOI 3 with PR8 at 8 h (**C**), or delNS1 at 16 h (**D**). EPZ was present all throughout the experiment in the EPZ-treated cells. The experiment was repeated twice, and representative images are shown. (**E**) Nuclear NF-κB translocation was measured by orthogonal projection image analysis and quantified in at least 200 cells/condition. The ratio was calculated by quantitation of the relative p65 intensity in the nucleus and the cytoplasm of each cell in all different conditions. Ratios are shown in dispersion graphs. ns *p* > 0.05; * *p* < 0.05; ** *p* < 0.01; *** *p* < 0.001.

Previous experiments showed that Dot1L inhibition decreased the recruitment of RIG-I to enriched mitochondrial fraction in influenza virus infected cells (Figure 3C). To analyze the possible role of NS1 on RIG-I-MAVS association, we compared that association in mitochondrial fractions of PR8 and delNS1-infected cells that were treated or not with the Dot1L inhibitor. Influenza virus infection and the efficacy of EPZ treatment were monitored by determining the accumulation of NP protein levels and total and H3K79 methylated histone 3 content (Figure 5A; Input) in the different conditions. Mitochondria purification was verified by the absence of MAVS in the P1 fraction (Figure 5A; P1) and its presence in the mitochondrial fraction (Figure 5A; Mito). Infection with PR8 increased the recruitment of RIG-I to the mitochondria as compared to uninfected control cells (MOCK). This association was higher in cells infected with delNS1 than in cells infected with the PR8 virus, in agreement with the increased antiviral response observed in delNS1 infected cells [39,40]. The recruitment of RIG-I to mitochondrial fractions was not modified by Dot1L inhibition in delNS1 infection.

We previously reported that NF-κB nuclear translocation is severely impaired by Dot1L inhibition upon influenza virus infection [5]. In addition, the aforementioned data suggested a role for NS1 in Dot1L regulation of the innate response against influenza infection. Thus, we asked whether NS1 was also required for EPZ-mediated inhibition of NF-κB translocation in response to viral infection. To this end, control or Dot1L-inhibited A549 cells were infected at MOI 3 with PR8 or delNS1 viruses, fixed at the peak of NP antigen expression (8 and 16 hpi, respectively) and the subcellular NF-κB localization was determined by confocal immunofluorescence microscopy (Figure 5B–D). The quantitation of the relative p65 intensity in the nucleus and cytoplasm is shown in Figure 5E. In non-infected cells (MOCK), NF-κB localization was entirely cytoplasmic and it was not affected by EPZ treatment (Figure 5B). The infection with PR8 and delNS1 provoked NF-κB nuclearization (Figure 5C–E) and, in agreement with our previous studies, the NF-κB nuclear translocation was inhibited by EPZ in the PR8 infected cells (Figure 5C,F). Dot1L inhibition did not significantly reduce NF-κB relocalization in delNS1 infection (Figure 5D,E). These data reinforce the notion that NS1 is an important determinant of the antiviral response when Do1L activity is inhibited.

### 3.6. Dot1L Inhibition Decreases the Expression of TRIM25 in Influenza Virus Infected Cells

Next, we examined the expression of cellular genes involved in the IFN response that were differentially modified during influenza virus infection in Dot1L inhibited A549 cells. In addition, the expression of influenza virus genes in untreated and EPZ-treated cells was also determined. The cells were left untreated or treated with 1 μM EPZ. Forty-eight hours later, the cells were infected with PR8 at MOI 3 and the total RNAs from duplicate cultures were isolated at 8 hpi and used for high-throughput sequencing as described in Section 2. EPZ was present all throughout the experiment in the EPZ-treated cells. In parallel, the accumulation levels of methylated H3K79 were monitored by Western blot and a quantitative colorimetric method based on anti-H3K79me2 antibody detection (Epigentek), as shown in [5]. A significant decrease of H3K79me2 levels was detected using both analyses, which indicated the efficiency of the EPZ treatment. As previously demonstrated, the EPZ treatment of A549 cells did not affect the cell viability over 48 h, as analyzed by MTT assay (data not shown) [41].

In agreement with the increased viral replication in Dot1L-infected cells, the increased expression of influenza virus RNAs was obtained in Dot1L downregulated cells (Figure S4). The relative expression values of the innate immune response-related mRNAs are shown in Table 1. The table displays data for the transcripts, showing significant alterations in influenza virus-infected cells left untreated (PR8/MOCK) or treated with EPZ (PR8-EPZ/MOCK-EPZ). In agreement with our previous report, EPZ treatment did not significantly interfere with ISG induction at this early time post-infection, as 16 h are required to restrict ISG induction by Dot1L inhibition [5]. A clear differential effect in *TRIM25* expression was observed. *TRIM25* expression was slightly increased by the infection in the absence of EPZ (although not statistically significant). However, its expression was downregulated in infected cells in the presence of EPZ.

A549 cells were untreated or treated with 1 μM of EPZ for 48 h and then infected with PR8 at MOI 3 for 8 h. The total RNA was used for RNA-sequencing and the list of proteins related to the innate immune response using a q value < 0.002 and log2 fold change > 1.5 is shown. (PR8/MOCK); genes whose expression changes in influenza virus infected cells, (PR8-EPZ/MOCK-EPZ); genes whose expression changes in infected and EPZ- treated cells. (*); q value, ns.

**Figure 6:**
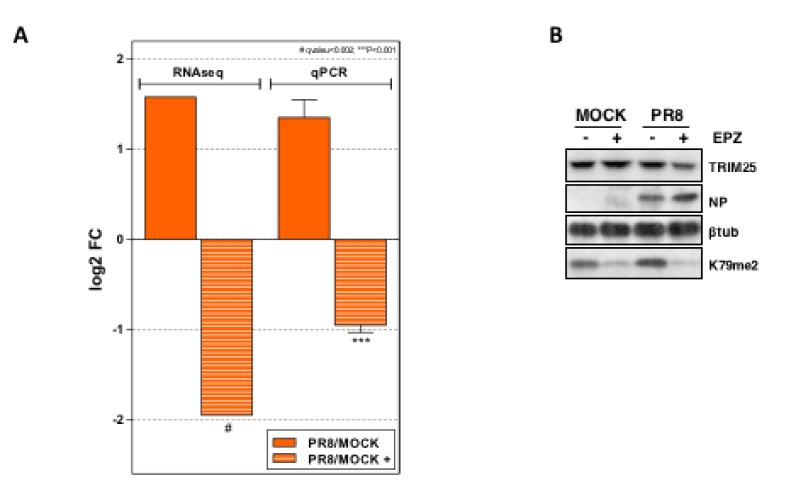
Changes of TRIM25 in control and Dot1L inhibited cells. (**A**) Comparison of the *TRIM25* expression from the RNA-seq and qRT-PCR data of A549 cells that were untreated or treated with EPZ (1 μM, 48 h) and then infected with PR8 at MOI 3 for 8 h. The differences between the untreated and treated cells were analyzed and are represented as the log2 fold change. Three technical replicates of three independent experiments for qRT-PCR detection were performed.. ns *p* > 0.05; * *p* < 0.05; ** *p* < 0.01; *** *p* < 0.001. (**B**) The total extracts of untreated or EPZ-treated cells, non-infected or infected with PR8 were used for Western blot analysis against the indicated proteins.

To verify this effect, we performed quantitative qPCR detection of *TRIM25* in the RNAs used for high-throughput sequencing (Figure 6A). An increase of *TRIM25* mRNA accumulation in the untreated and infected cells was found. Moreover, a clear decrease in the Dot1l inhibited-infected cells was also observed, which confirms the results obtained in the RNA seq approach. In addition, TRIM25 protein levels were analyzed in the infected A549 cells untreated or treated with EPZ. The EPZ treatment was monitored by Western blot using an antibody against H3K79me2, the target of Dot1L. According to the mRNA data, reduced accumulation of TRIM25 was observed in Dot1L inhibited PR8-infected cells (Figure 6B). These results may account for the reduced responsiveness of EPZ-treated cells against viral infection. An increased expression of *TRIM25* in uninfected A549 cells EPZ-treated analyzed by high-throughput RNA sequencing, which was confirmed by qPCR, was previously observed [8]. Collectively, the data indicate that the *TRIM25* gene is a target for Dot1L histone methylase.

### 3.7. Overexpression of TRIM25 Abolishes the Effect of Dot1L Inhibition on Interferon Signaling

To evaluate whether TRIM25 could counteract the reduction of the antiviral response mediated by Dot1L inhibition, TRIM25 overexpression experiments were carried out. First, we tested the effect of TRIM25 overexpression in the IFN-β reporter assay. Control or EPZ-treated A549 cells were transfected with the pIF-LucTer and increasing amounts of the TRIM25-expressing plasmid (pCDNA3.0-HA-TRIM25; 10, 20, or 50 ng per 96-well) and 24 h later the luciferase activity was measured. A dose dependent increase in the luciferase activity was observed upon TRIM25 overexpression (Figure S5). Moreover, the EPZ treatment decreased the luciferase activity in the 10 and 20 ng of TRIM25 transfected plasmid conditions but was unable to do so at the highest dose of TRIM25 plasmid (Figure S5).

**Figure 7:**
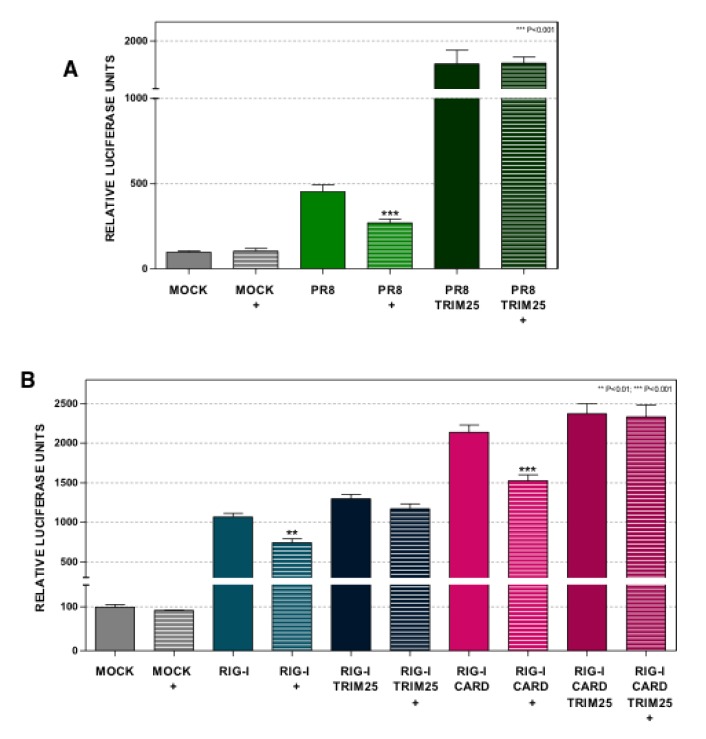
The overexpression of TRIM25 counteracts the effect of Dot1L inhibition on interferon signaling. (**A**) A549 cells were left untreated (MOCK) or treated with 1 μM EPZ (+) for 48 h. Then the cells were transfected with pIF-LukTer alone (MOCK), or together with a TRIM25 expressing plasmid (TRIM25) or a control plasmid. After 24 h, the cells were infected with influenza virus PR8 (PR8 and PR8 TRIM25) at MOI 1 and the luciferase activity was evaluated at 16 hpi. (**B**) A549 cells were left untreated (MOCK) or treated with 1 μM EPZ (+) for 48 h. Then the cells were transfected with pIF-LukTer alone (MOCK), or together with plasmids expressing RIG-I (RIG-I), or RIG-I and TRIM25 (RIG-I TRIM25), or RIG-I 2CARDs (RIG-I CARD), or RIG-I 2CARDs and TRIM25 (RIG-I CARD TRIM25). The luciferase activity was evaluated 16 h later. The luciferase activity was normalized by Renilla luciferase and it was expressed relative to that of the untreated MOCK condition. Three technical replicates of three independent experiments were analyzed. ns *p* > 0.05; * *p* < 0.05; ** *p* < 0.01; *** *p* < 0.001.

Next, the EPZ-treated or untreated A549 cells were transfected with the pIF-LukTer plasmid in the presence or absence of 20 ng of the TRIM25-expressing plasmid, and 24 h later, the transfected cells were infected with influenza PR8 virus at MOI 1. The luciferase activity was then measured at 16 hpi (Figure 7A). The overexpression of TRIM25 increased the IFN-β promoter activation in the infected cells. Importantly, the TRIM25-mediated enhancement of the innate response was unresponsive to EPZ. The data suggests that TRIM25 overexpression is sufficient to compensate the reduction of the innate response against the influenza virus mediated by Dot1L inhibition.

To further evaluate the role of TRIM25 in the control of the RIG-I signaling mediated by Dot1L, the control or Dot1L-inhibited A549 cells were transfected with pIF-LukTer and with plasmids that express RIG-I or the RIG-I-2CARDs, together with 20 ng of the TRIM25-expressing plasmid (Figure 7B). The expression of TRIM25 counteracted the reduced luciferase activity upon Dot1L inhibition also in the RIG-I or RIG-I 2CARDs expressing cells.

### 3.8. Expression of A K63 Ubiquitination Defective RIG-I Mutant Abolishes the Effect of Dot1L Inhibition on Interferon Signaling

**Figure 8:**
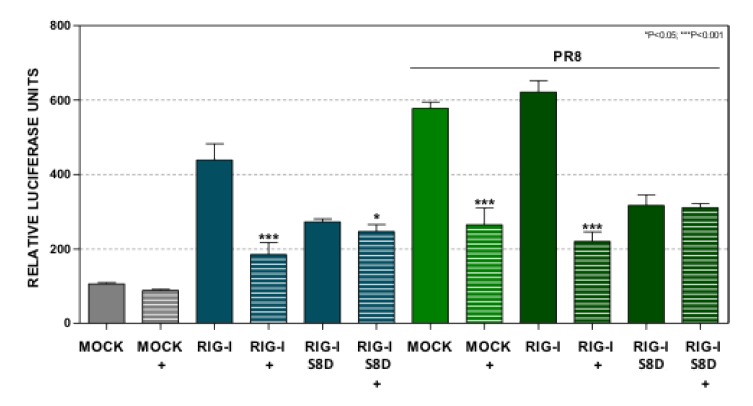
INF-β driven expression triggered by a mutated RIG-I at Ser 8 is not regulated by Dot1L. A549 cells were left untreated (MOCK) or treated with 1 μM EPZ (+) for 48 h. Then the cells were transfected with pIF-LukTer alone (MOCK), or together with plasmids expressing the wild type RIG-I (RIG-I), or a mutated form of RIG-I with a Ser to Asp substitution at position 8 of the CARDS domains (RIG-I S8D). The luciferase activity was evaluated at 16 hpt. The cells were left uninfected or infected with PR8 at MOI 3 for 8 h. Luciferase activity was normalized by Renilla luciferase and it was expressed relative to that of the untreated MOCK condition. Three technical replicates of three independent experiments were analyzed. ns *p* > 0.05; * *p* < 0.05; ** *p* < 0.01; *** *p* < 0.001.

RIG-I dephosphorylation is required for TRIM25-mediated specific lysine 63 ubiquitination to elicit an antiviral response [21]. The control or EPZ-treated A549 cells were transfected with pIF-LucTer together with plasmids expressing wild type RIG-I or a mutated RIG-I containing a Ser 8 to Asp substitution in the 2CARDS domain (RIG-I S8D). The transfected cells were left uninfected or infected with PR8 at MOI 1 for 16 hpi (PR8) (Figure 8). This phosphomimetic mutation reduces the binding of RIG-I to TRIM25, decreasing the RIG-I ubiquitination and thereby diminishing the binding to the CARD domain with MAVS [21]. Treatment with EPZ reduced the IFN-β promoter stimulation in cells expressing wild type RIG-I uninfected or infected with influenza virus and in untransfected-infected cells. In contrast, Dot1L inhibition did not modify the luciferase activity in cells expressing the phosphorylation RIG-I mutant in any of the experimental conditions. These results support that Dot1L control of IFN signaling may rely on the TRIM25 association with the 2CARDS domains of RIG-I.

## 4. Discussion

Dot1L is a histone methylase, which modifies exclusively lysine 79 of histone 3 (H3K79), a lysine located within the globular domain of histone H3 that can be mono-, di-, and trimethylated by Dot1L [6]. Dot1L has been implicated in transcriptional activation and accordingly, methylated H3K79 has been constantly found within the bodies of the transcribing genes in different species, including the human genome [7,42]. Infection with human cytomegalovirus, a virus bearing a large double-stranded DNA genome that becomes associated with host cell histones [43], increases Dot1L expression and H3K79me2, which overlaps with the beginning of viral replication [44]. However, there are no reports on the role of Dot1L in RNA virus infections.

While characterizing the epigenetic changes induced by the influenza virus in the host cell, we found a prominent increase at the mono- and dimethylated lysine 79 of histone 3. Subsequent studies using a chemical inhibitor of Dot1L or specific Dot1L silencers, showed that the downregulation of Dot1L increased influenza virus replication through a decrease of the IFN-β signaling pathway [5]. Conversely, upregulation of Dot1L reduced influenza virus replication [8]. The augmented H3K79 methylation triggered by the influenza virus may increase the IFN-β pathway stimulation and the antiviral response and would represent a host cell defense response to the infection.

**Figure 9:**
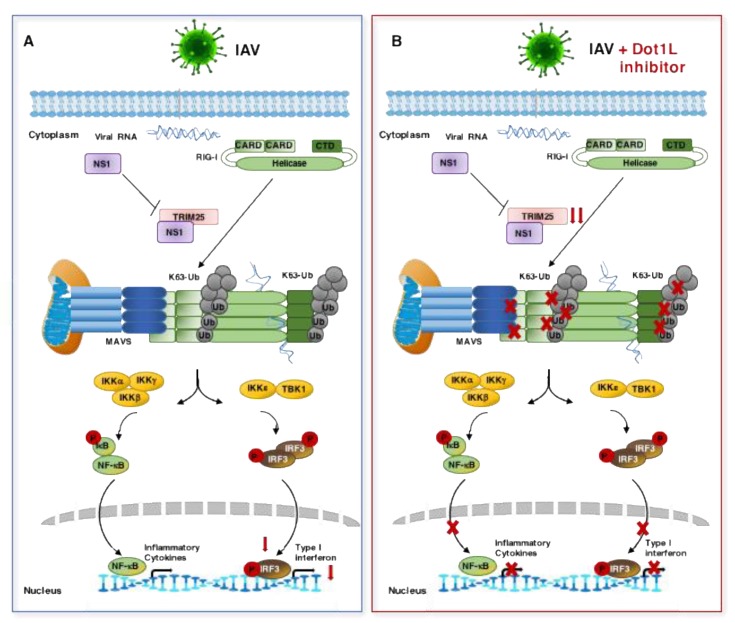
The effects of Dot1L inhibition in influenza virus infected cells. (**A**) Influenza virus infection stimulates the production of inflammatory cytokines and type I interferon through the RIG-I signaling pathway. The NS1 protein interacts with TRIM25 and partly counteracts IFN signaling. (**B**) In Dot1L-inhibited and infected cells, there is a decrease of *TRIM25* expression that elicited impaired RIG-I ubiquitination, RIG-I-MAVS association, and NF-κB and IRF3 activation. The production of inflammatory cytokines and type IFN are reduced, which decreases the antiviral response activating virus replication.

The RNA analysis of genes involved in the interferon signaling pathway indicated that influenza virus infection stimulates *TRIM25* expression, which in turn markedly decreases when infected human alveolar epithelial cells are treated with a Dot1L inhibitor (Table 1, Figure 6). Characterization of the immune response induced by different pathogens in murine alveolar macrophages infected at high MOI [40], showed a higher induction of *TRIM25* expression in delNS1 infected cells compared with PR8 infection (data not shown). TRIM25-mediated ubiquitination stimulates RIG-I tetramerization, allowing RIG-I-MAVS interaction, which triggers the induction of the innate immune response [45,46,47]. The influenza virus expresses the NS1 protein, a multifunctional protein capable of counteracting the antiviral response [37]. The interaction of NS1 with RIG-I has been reported [48]. In addition, NS1 interacts with TRIM25 impairing the formation of the RIG-I-MAVS complexes [35]. The C-terminal domain of NS1 appears to be involved in NS1-TRIM25 interaction as C-terminal mutants do not bind TRIM25 and do not block RIG-I ubiquitination mediated by TRIM25 [49,50,51]. In contrast to PR8 infection, viral replication (Figure 4A), the activation of IFN-β-driven expression (Figure 4B), RIG-I-MAVS association (Figure 5A), and NF-kB nuclearization (Figure 5D,E) are not affected by Dot1L inhibition in delNS1 infection. These data agree with the described role of the NS1 protein that would interact with TRIM25, competing with the TRIM25-RIG-I association and decreasing the antiviral response (Figure 9A). In the infected and Dot1L-inhibited cells, decreased levels of TRIM25 occur, which would diminish RIG-I ubiquitination, RIG-I-MAVS association, RIG-I signaling, and the antiviral response and consequently increases viral replication (Figure 9B). As described, in cells infected with the delNS1 virus, increased antiviral response occurs, partly mediated by the absence of competitor TRIM25-RIG-I associations. In this situation, decreased levels of TRIM25 by Dot1L inhibition may generate small changes in RIG-I-MAVS interaction with slight changes in the antiviral response.

Different RNA viruses have developed similar strategies focused on TRIM25 impairment to counteract the antiviral response. The V protein of the Sendai virus [52] also competes with TRIM25-RIG-I association. On the other hand, vesicular stomatitis virus infection downregulates NLRP12 expression, a protein that also associates with TRIM25 [53]. According to the role of TRIM25 as a check point of the innate immune induction, the decreased levels produced by Dot1L downregulation stimulate influenza and vesicular stomatitis virus replication [5] and reduce the induction of type I interferon in influenza, vesicular stomatitis, and Sendai virus infected cells (Figure 1). In addition, TRIM25 overexpression restores the stimulation of type I IFN in Dot1L inhibited-influenza virus infected cells or in cells expressing different intermediates of the RIG-I signaling pathway (Figure 7).

Dot1L was found to be associated to protein complexes that contain Mixed Lineage Leukemia (*MLL*) fusion genes as a consequence of *MLL* translocations [6,54]. Many different partners have been described to fuse to the protein complexes containing the *MLL* gene. Many of them are factors involved in transcriptional regulation, such as Dot1L, which is frequently found in those complexes [54,55]. The host-factors relevant for infection, such as TRIM25, seem to be a target for Dot1L modulation even in uninfected cells [8]. In agreement with that, an important number of the frequent treatment-emergent adverse events that may be related with infection were reported in Phase 1 clinical trials of MLL-r patients treated with pinometostat (EPZ) [56]. The ability of Dot1L to regulate IFN signaling, supports an important and general role of Dot1L in the control of pathogen infections. Changes in H3K79 methylation during infection may modulate the access of transcriptional complexes to the genes involved in the control of the antiviral response and, thus, changes in the chromatin structure need to be considered as additional host-mechanisms for the control of RNA viruses.

## Figures and Tables

**Table 1:** Cellular genes involved in the IFN response differentially modified during influenza virus infection in Dot1L inhibited A549 cells.

Gene Name	Gene ID	Description	PR8/MOCK Log2FC	PR8-EPZ/MOCK-EPZ Log2FC
OAS2	4938	2′-5′-oligoadenylate synthetase 2	40.32	39.77
IFNL1	282618	Interferon lambda1	39.43	39.20
IFNL2	282616	Interferon lambda2	39.18	38.81
IFNL3	282617	Interferon lambda3	38.66	38.52
IL17F	112744	Interleukin 17F	38.53	39.00
MX2	4600	MX dynamin like GTPase2	38.53	38.43
IL13	3596	Interleukin 13	37.46	36.96
IFNB1	3456	Interferon beta 1	36.79	36.98
IL17C	27189	Interleukin 17C	36.49	35.07
IRF7	3665	Interferon regulatory factor 7	35.35	36.29
IFIT2	3433	Interferon induced protein with tetratricopeptide repeats 2	7.45	6.97
MX1	4599	MX dynamin like GTPase1	7.22	6.52
OASL	8638	2′-5′-oligoadenylate synthetase like	7.00	7.56
IFI6	2537	Interferon induced protein with tetratricopeptide repeats 6	6.58	6.28
IFI44	10561	Interferon induced protein with tetratricopeptide repeats 44	6.39	6.34
ISG15	9636	ISG15 ubiquitin-like modifier	6.31	6.48
IFIT1	3434	Interferon induced protein with tetratricopeptide repeats 1	5.79	6.07
IFIT3	3437	Interferon induced protein with tetratricopeptide repeats 3	5.62	4.96
TRIM22	10346	Tripartite motif containing 22	3.52	2.94
TRIM21	6737	Tripartite motif containing 21	2.51	2.49
OAS1	4938	2’-5’-oligoadenylate synthetase 1	2.47	2.51
TRIM56	81844	Tripartite motif containing 56	1.99	1.44
IL15	3600	Interleukin15	−2.16	−2.27
**TRIM25**	**7706**	**Tripartite motif containing 25**	1.58 *	**−1.95**
TRIM68	55128	Tripartite motif containing 68	−1.76	−2.18
TRAF2	7186	TNF receptor associated factor 2	−1.6	−1.99

Log2FC >1.5 and <-1.5, q value < 0.002. (*); *q* value, ns.

## Supplementary Materials

The following are available online at https://www.mdpi.com/2073-4409/9/3/732/s1, Figure S1: MDA5 does not play a pivotal role in the Dot1L-mediated regulation of IFN pathway, Figure S2: Dot1L inhibition reduces MAVS aggregation in influenza virus infected cells. Figure S3. Dot1L modulates MAVS activation, RIG-I-MAVS association and antiviral response mediated by RIG-I sensor. Figure S4. Effect of Dot1L inhibition in the influenza virus RNAs expression. Figure S5. Effect of TRIM25 overexpression in the IFN-β reporter assay.

## Computer Code

All RNA-seq data are deposited in the NCBI Sequence Read Archive (SRA). Those belonging to MOCK samples are deposited under BioProject identifier PRJNA562125 (SRP124845) and the rest of samples are deposited under BioProject identifier PRJNA580198 (SRP227330). Studies: https://dataview.ncbi.nlm.nih.gov/object/PRJNA562125?reviewer=bj3spmb63srg6bp6ab4j3ka78g and https://dataview.ncbi.nlm.nih.gov/object/PRJNA580198?reviewer=3ljaeoj3phi52dq5ckpc5u1pc1.
