# Supplementary material for: Interferon-β Stimulation Elicited by the Influenza Virus Is Regulated by the Histone Methylase Dot1L through the RIG-I-TRIM25 Signaling Axis
Source: Cells. 2020 Mar 16;9(3):732. doi: 10.3390/cells9030732 (PMC7140698; doi:10.3390/cells9030732)
Supplement: Supplementary file 1 [file cells-09-00732-s001.pdf]

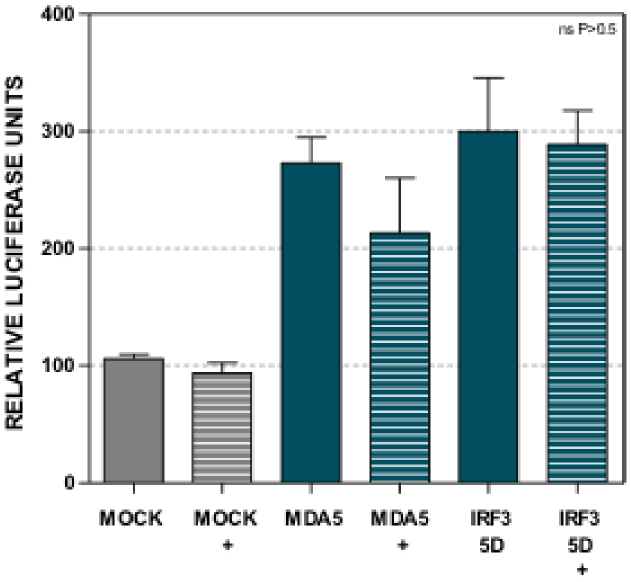

Figure S1. MDA5 does not play a pivotal role in the Dot1L-mediated regulation of IFN pathway. A549 cells were left untreated (MOCK) or treated with 1 μM EPZ (+). 48 h later the cells were transfected with pIF-LukTer alone (MOCK), or together with plasmids expressing MDA5, or a mutant form of IRF3 (IRF3 5D) and the luciferase activity was evaluated at 16 hpt. Luciferase activity was normalized by Renilla luciferase and it was expressed relative to that of untreated control condition. Three technical replicates of three independent experiments were analyzed. ns P>0.05; *P<0.05; **P<0.01; ***P<0.001.

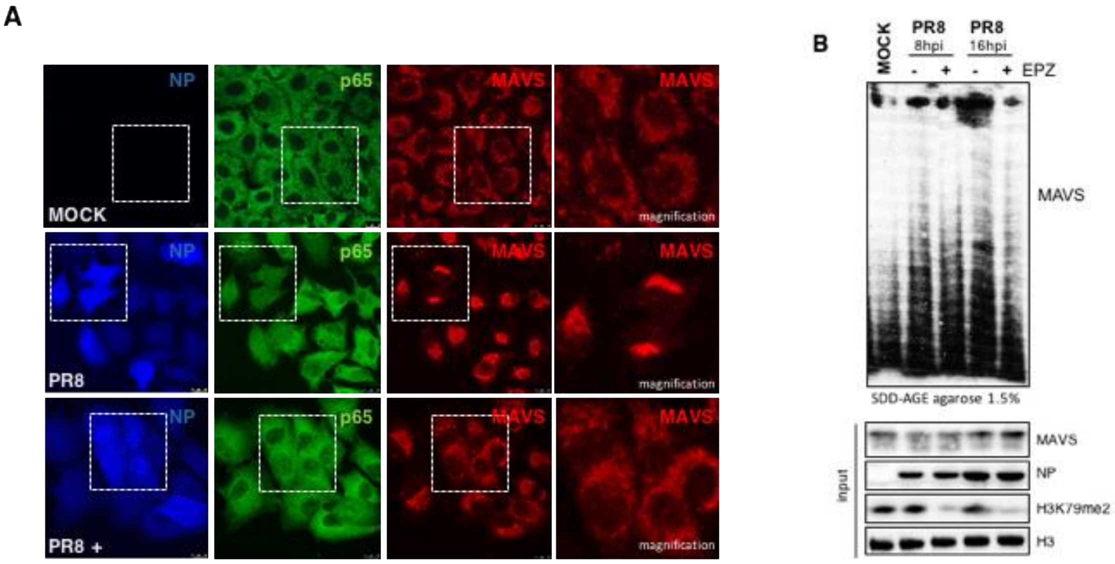

Figure S2. Dot1L inhibition reduces MAVS aggregation in influenza virus infected cells. (A); control or EPZ-treated cells (+), during 48h were left uninfected (MOCK) or infected with PR8 at MOI 3 and processed for immunofluorescence using anti-NP, anti-p65 and anti-MAVS antibodies. (B); A549 cells were plated in the presence or absence of EPZ 1μM and the cells were left uninfected (MOCK) or infected with PR8 during 8 h or 16 h. Samples were analyzed by SDD-AGE (upper part) to analyze MAVS polymers using anti-MAVS antibody. Lower part; the presence of the relevant proteins in the input was analyzed by Western blot.

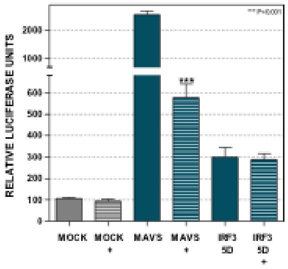

	RELATIVE LUCIFERASE UNITS	LUCIFERASE UNITS	*RENILLA* UNITS
MOCK	110	486970	4427
	103	422815	4105
	105	231630	2206
MOCK +	91	671332	7338
	86	681513	7936
	103	996948	9637
MAVS	2532	4993735	1972
	2400	6144000	2560
	2680	7766640	2898
MAVS +	551	3012595	5468
	650	3727750	5735
	530	2668550	5035
IRF35D	350	957950	2737
	289	126293	437
	260	567580	2183
IRF35D +	256	520192	2032
	300	987300	3291
	310	868000	2800

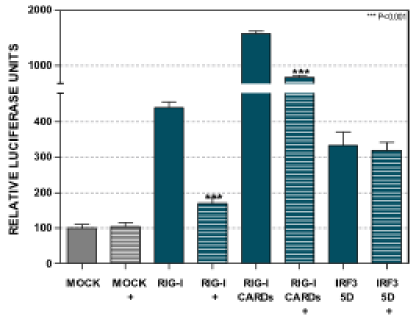

	RELATIVE LUCIFERASE UNITS	LUCIFERASE UNITS	*RENILLA* UNITS
MOCK	110	306020	2782
	100	187700	1877
	90	218790	2431
MOCK +	91	202735	2216
	116	272656	2353
	103	337040	3258
RIG-I	435	3414750	7850
	457	3092062	6766
	427	3130764	7332
RIG-I +	176	1849584	10509
	180	1962360	10902
	154	1376606	8939
RIG-I CARD	1537	15711626	10223
	1630	22066940	13538
	1570	13724940	8742
RIG-I CARD +	811	11122226	13711
	798	11651598	14601
	736	4495488	6108
IRF35D	350	561400	1604
	289	438702	1518
	360	132480	368
IRF35D +	345	521985	1513
	300	657300	2191
	310	608220	1962

Figure S3. Dot1L modulates MAVS activation, RIG-I-MAVS association and antiviral response mediated by RIG-I sensor. (A); A549 cells were left untreated (MOCK) or treated with 1 μM EPZ (+). 48 h later the cells were transfected with pIF-LukTer alone (MOCK), or together with plasmids expressing MAVS (MAVS), or a mutant form of IRF3 (IRF3 5D) and the luciferase activity was evaluated at 16 hpt. (B); Control or EPZ-treated cells were transfected with pIF-LukTer alone (MOCK), or together with plasmids expressing RIG-I (RIG-I), or RIG-I CARDs (RIG-I CARDS), or a mutant form of IRF3 (IRF35D) and the luciferase activity was evaluated at 16 hpt. Luciferase activity was normalized by *Renilla* luciferase. MOCK condition without EPZ treatment was set as 100%. The relative luciferase units obtained in each condition are represented below each graph. Three technical replicates of three independent experiments were analyzed. ns P>0.05; *P<0.05; **P<0.01; ***P<0.001.

Gene Name	Gene_ID	Description	PR8/MOCK Log2FC	PR8/MOCK EPZ Log2FC	PR8-EPZ/PR8 FC
**PB2**	956536	Influenza A polymerase PB2	10.49	12.48	3.97
**PB1**	956534	Influenza A polymerase PB1	10.56	11.49	1.90
**PA**	956535	Influenza A polymerase PA	10.53	11.51	1.97
**NP**	956531	Influenza A nucleoprotein	10.41	11.38	1.95
**HA**	956529	Influenza A haemagglutinin	10.49	11.31	1.76
**NA**	956530	Influenza A neuraminidase	10.33	10.29	0.97
**NS1**	956533	Influenza A nonstructural protein 1	10.56	11.82	2.39
**NEP**	956532	Influenza A nonstructural protein 2	10.56	11.82	2.39

qvalue <0.002 and log2 fold change >1.9

Figure S4. Effect of Dot1L inhibition in the influenza virus RNAs expression. A549 cells were untreated or treated with EPZ 1 μM for 48 h and then infected with PR8 at MOI 3. Total RNA extracted at 8 hpi was used for RNA-sequencing. Viral RNAs are represented using a qvalue <0.002 and log2 fold >1.9. (PR8/MOCK); expression of viral RNAs in influenza virus infected cells, (PR8-EPZ/MOCK-EPZ); expression of viral RNAs in infected and EPZ- treated cells.

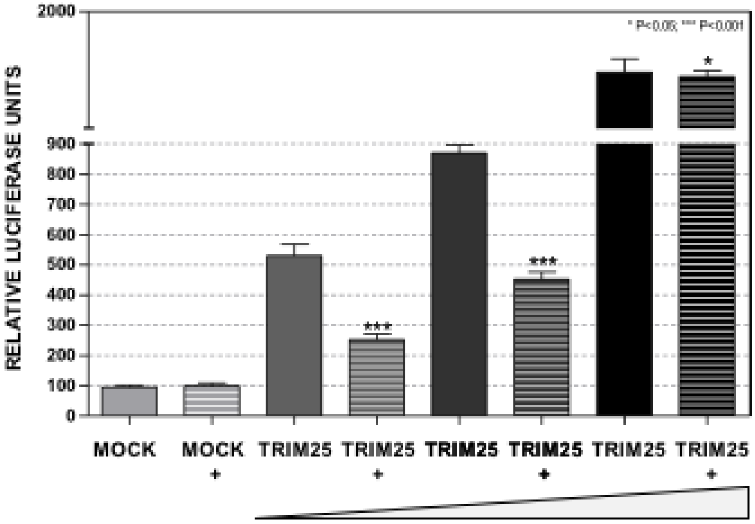

Figure S5. Effect of TRIM25 overexpression in the IFN-β reporter assay. Control or EPZ-treated A549 cells were transfected with pIF-LucTer and increasing amounts of the plasmid expressing TRIM25 (pCDNA3.0-HA-TRIM25; 10, 20 or 50 ng per 96 well) and luciferase activity was measured 24 h later. Luciferase activity was normalized by *Renilla* luciferase. MOCK condition without EPZ treatment was set as 100%. Three technical replicates of three independent experiments were analyzed. ns P>0.05; *P<0.05; **P<0.01; ***P<0.001.
